# Small variable segments constitute a major type of diversity of bacterial genomes at the species level

**DOI:** 10.1186/gb-2010-11-4-r45

**Published:** 2010-04-30

**Authors:** Fabrice Touzain, Erick Denamur, Claudine Médigue, Valérie Barbe, Meriem El Karoui, Marie-Agnès Petit

**Affiliations:** 1 INRA, UMR1319, Micalis, Bat 222, Jouy en Josas, 78350, France; 2 INSERM U722 and Université Paris 7, Faculté de Médecine, Site Xavier Bichat, Paris, 75018, France; 3 CNRS-UMR 8030 & CEA/IG/Genoscope, Laboratoire d'Analyses Bioinformatiques en Génomique et Métabolisme (LABGeM), rue Gaston Crémieux, Evry, 91057, France; 4 CEA, Institut de Génomique, Genoscope, rue Gaston Crémieux, Evry, 91057, France

## Abstract

**Background:**

Analysis of large scale diversity in bacterial genomes has mainly focused on elements such as pathogenicity islands, or more generally, genomic islands. These comprise numerous genes and confer important phenotypes, which are present or absent depending on strains. We report that despite this widely accepted notion, most diversity at the species level is composed of much smaller DNA segments, 20 to 500 bp in size, which we call microdiversity.

**Results:**

We performed a systematic analysis of the variable segments detected by multiple whole genome alignments at the DNA level on three species for which the greatest number of genomes have been sequenced: *Escherichia coli*, *Staphylococcus aureus*, and *Streptococcus pyogenes*. Among the numerous sites of variability, 62 to 73% were loci of microdiversity, many of which were located within genes. They contribute to phenotypic variations, as 3 to 6% of all genes harbor microdiversity, and 1 to 9% of total genes are located downstream from a microdiversity locus. Microdiversity loci are particularly abundant in genes encoding membrane proteins. In-depth analysis of the *E. coli *alignments shows that most of the diversity does not correspond to known mobile or repeated elements, and it is likely that they were generated by illegitimate recombination. An intriguing class of microdiversity includes small blocks of highly diverged sequences, whose origin is discussed.

**Conclusions:**

This analysis uncovers the importance of this small-sized genome diversity, which we expect to be present in a wide range of bacteria, and possibly also in many eukaryotic genomes.

## Abstract 2

Comparison of all available genomes of three bacterial species suggests that a large part of genome diversity is contributed by short regions.

## Background

The availability of bacterial genome sequences for closely related strains within a species and software dedicated to multiple genome alignments allow for a novel perspective of bacterial genetic diversity [1-3]. Use of these aligners has led to the notion that bacterial species share a DNA backbone common to all strains interrupted by variable segments (VSs) that are specific to a subset of the aligned strains [4-6]. The most studied category of VSs are genomic islands, which are defined by Vernikos and Parkhill as horizontally acquired mobile elements of limited phylogenetic distribution [7]. These islands are of a large size (30 to 100 kb), and often encode genes critical for pathogenesis [8]. Their integration into genomes presumably occurs by site-specific recombination. Genomic islands may then diffuse from strain to strain by homologous recombination [9]. Where known, horizontal transfer of islands occurs either by mobilization through bacteriophages, such as in *Staphylococcus aureus *[10,11] or by conjugation, using transfer origins located either outside or inside the island [9,12,13]. Informatic tools have been developed to detect such islands in genomes [14-16]. A second category of VSs of large size involves temperate bacteriophages, or phage remnants. Like genomic islands, they enter the bacterial chromosome by site-specific recombination. Informatic tools to predict these elements have flourished in the past few years [17-19]. Recently, a new class of large variable elements has been characterized with the clustered, regularly interspaced short palindromic repeats (CRISPR), in which repeats alternate with short DNA segments of plasmid or bacteriophage origin. These regions confer phage or plasmid immunity [20,21] by mechanisms that remain to be understood. Databases for these elements are available [22,23]. Transposons and insertion sequences (ISs) also contribute to VSs when closely related genomes are compared, and their size is small compared to the first two types of elements (a few hundred base pairs to a few kilobases). These elements move within a given genome by transposition. A reference website allowing their classification exists [24], and two strategies for automated IS detection have been described [25,26]. Finally, the smallest kind of VS (with a = 20 bp threshold) expected to be present when genomes are aligned are the minisatellites, composed of small tandem repeats that are commonly used for strain typing. Websites allowing their recognition are available [27-29]. A special category of such repeats are the 'small dispersed repeats', some 20 bp long and tandemly repeated in various copy numbers in genomes, which might be mobile [29]. The *Escherichia coli *genomes contain a family of such elements, called palindromic units (PUs; 30 to 37 bp), which are palindromic and intergenic, and often combined in clusters [30].

DNA recombination and mutagenesis are the sources of respectively large and small scale genetic diversity in genomes. In a broad sense, recombination designates all events that reshuffle DNA sequences. This reshuffling can have two opposite effects: either it homogenizes DNA sequences (a process called DNA conversion), or it provokes the abrupt loss, acquisition or translocation of genetic information, and therefore brings in diversity. A wide range of artificial genetic systems have been set up in the past decades to study recombination at the molecular level in bacteria and to determine the frequencies of its occurrence. Among the three main categories of recombination events, site-specific recombination is highly efficient; for example, recombination can occur in 100% of cells in an engineered site-specific recombination assay [31]. However, this class of events is limited by its specialization, as it requires a dedicated enzyme (whose expression is usually regulated) and its cognate site. The next most efficient bacterial system is homologous recombination; for example, an estimated 10^-4 ^of a non-stressed cell population recombined 1-kb-long tandem repeats present in the chromosomes of *Salmonella typhimurium *[32], *E. coli *[33], *Bacillus subtilis *[34] and *Helicobacter pylori *[35]. These events usually rely on RecA, an ubiquitous enzyme that catalyzes homologous DNA pairing. Homologous recombination is not sequence-specific, and its efficiency is proportional to the length of homology shared by the recombining molecules. High proportions of recombinants are scored during DNA conjugation (up to 10%), where several hundred-kilobase-long DNA segments enter the cell [36], and during natural DNA transformation [37]. Finally, illegitimate recombination is the least efficient mode of recombination, with events occurring in approximately 10^-8 ^of a given cell population [38,39]. It includes events that join DNA segments not sufficiently homologous for RecA pairing, nor involved in site-specific recombination. Illegitimate recombination events are attributed to errors of enzymes that deal with DNA, such as DNA polymerases [40-42], RNA polymerases [43], repair enzymes, or topological enzymes (for reviews, see [44,45]). Interestingly, the non-homologous end joining type of illegitimate recombination, which involves dedicated enzymes and has a pre-eminent role in eukaryotes, is almost absent in prokaryotes, except in a few species such as *Mycobacterium tuberculosis *[46,47] and *B. subtilis*, where it contributes to spore germination and resistance to desiccation [48,49].

To date, no correlation exists between experimental DNA recombination studies and comparative genomic analyses. Indeed, molecular analyses usually focus on a single type of event (for examples, see [34,38,42]) without considering its frequency compared to those of other events that occur in the natural history of bacterial genomes. It is conceivable that the least efficient - that is, illegitimate recombination - is the major contributor in shaping bacterial genomes. Comparative genomic analyses offer the possibility to examine genome diversity globally, but most studies usually concentrate on just a single class of VSs. One exception involves a systematic analysis of all VSs of more than 10 bp present on two very closely related *S. aureus *genomes [50]. Among 27 VS sites, this study revealed a pre-eminence of illegitimate events over other classes of recombination, and raises questions of whether this observation can be generalized to more diverse genomes, and to other species.

In this report, we performed multi-strain alignments in three very different species to make a global assessment of bacterial diversity. Our aim was to understand the kind of molecular events that shaped present day genomes, and to determine the features of recombination. Our main finding is that short VSs (20 to 500 bp long) are highly frequent in genomes and reside often within genes. Such VSs are sometimes referred to as indels, but our multigenome analysis shows that only a minority of them originates effectively from an insertion or a deletion; we therefore designated them collectively by the broader term of 'microdiversity'. This study uncovers the numerical importance of microdiversity, predicts the pre-eminence of illegitimate recombination as the mechanism generating it, and highlights the existence, among microdiversity, of highly diverged blocks.

## Results

### Strain choice

**Figure 1:**
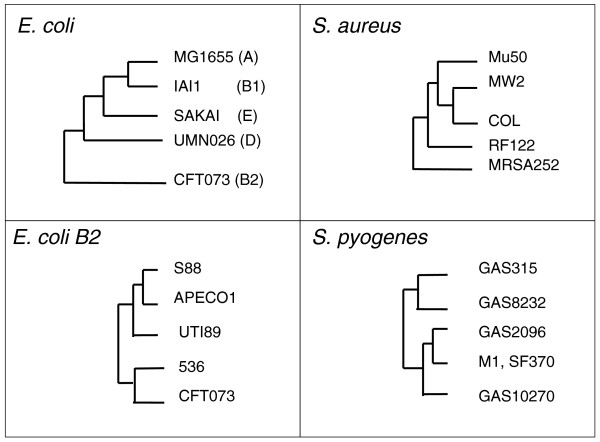
**Neighbor joining trees based on genomic MUMi distances of the strains selected for the five-genome alignments**.

*E. coli*, *S. aureus *and *Streptococcus pyogenes *were selected to examine intra-species diversity at the genome level, as they are the three species with the greatest number of available genome sequences. Members of each species are known pathogens, but otherwise they have very diverse characteristics: *E. coli *is a Gram-negative bacterium that lives both in the digestive tract of warm blooded animals and in water, while *S. aureus *and *S. pyogenes *are Gram-positive species that respectively colonize the nose, and skin and throat of mammals. Unlike the two other species, *S. pyogenes *is an obligate fermenting bacterium. Five genomes representative of each of these species were selected such that each member of the set was as distant as possible from all others (see Materials and methods). The *E. coli *species is particularly diverse, and phylogenetic studies led to the conclusion that a branch of this species, the B2 phylogenetic group, behaves as a subspecies [51,52]. Moreover, the comparative study of 20 *E. coli *genomes identified a substantial set of genes that are unique to the B2 group [53]. We therefore analyzed a set of five *E. coli *B2 genomes as a group, in addition to the genome set representative of the *E. coli *species. Neighbor joining trees derived from a new genomic distance called MUMi (see Materials and methods) [54] were calculated for the four strain sets (Figure 1). The *E. coli *MUMi tree was congruent with the phylogenetic tree reconstructed from the *Escherichia *core genome genes [53]. As for the *S. aureus *and *S. pyogenes *sets, reliable phylogenetic trees derived from the concatenated core genome of the species are not yet available to our knowledge, but our previous results suggest that the MUMi trees should be good approximations of phylogenetic trees [54].

To complete the five genomes analyses, alignments involving a maximum number of genomes were also analyzed using 25, 11 and 12 genomes for *E. coli*, *S. aureus *and *S. pyogenes*, respectively. Trees of the strains used are shown in Additional file 1.

### Alignments and definition of the variable segments

Complete multiple genome aligners provide general outlines of colinear regions among the genomes, as well as the set of identical anchors (short DNA fragments) shared by all genomes. Out of these data, complete alignments can be defined precisely using a post-treatment step, so as to attribute which parts of the genomes belong to the common backbone DNA, and which parts are VSs (see Materials and methods). MOSAIC [55] is a database offering such completely refined alignments for bacterial genomes at the intra-species level, using either MGA or MAUVE as entry points for the post-treatment step. We have shown previously [4,5] that it is possible to use robust criteria to delineate VSs: if in a part of the alignment at least two DNA segments differ by more than 24% at the nucleotide level, or if the alignment includes a gap of at least 20 nucleotides, all segments of this part of the alignment are labeled as VSs. Further details on these parameter choices are given in the Materials and methods and in Additional file 2.

VSs are defined here as DNA segments with a minimum length of 20 bp, and that differ from one another at a given position of the alignment. The cutoff chosen to decide that two VSs differ from one another is largely above the average pairwise nucleotide diversity between orthologous genes, which usually does not exceed 5% at the intra-species level in bacteria. As a consequence, in this analysis, all sequences having point mutations corresponding to the intra-species vertical divergence, as well as small indels, are classified as the backbone and are not considered.

The main characteristics of the alignments are presented in Table 1. While the *E. coli *strains were, as expected, more distantly related to one another than strains of the other sets [54] (see the longer branches in Figure 1, and maximal MUMi values in Table 1), the B2*E. coli*, *S. pyogenes *and *S. aureus *sets had similar 'tree depth', suggesting that these three sets diverged during similar evolutionary time scales.

**Table 1:** Characteristics of the four whole-genome alignments, involving five strains each

	*E. coli*	*E. coli *B2	*S. aureus*	*S. pyogenes*
Median genome size (Mb)	5.2	5.2	2.8	1.8
Maximal MUMi distance	0.3	0.156	0.197	0.175
Coverage^a^	72.7%	83.5%	84.5%	83.5%
Percent identity of backbone	98.05%	99.43%	98.73%	99.18%
Total number of loci^b^	1,037	539	768	344
Number of microdiversity loci	640	370	556	250
Median size of VS (bp)^c^	93	68	78	61

^a^Proportion of the genome included in the backbone (average). ^b^Positions in the alignment where the backbone is interrupted by at least one variable segment (VS).

### VSs are abundant, short in size, and, for the most part, different from previously reported variable elements

**Figure 2:**
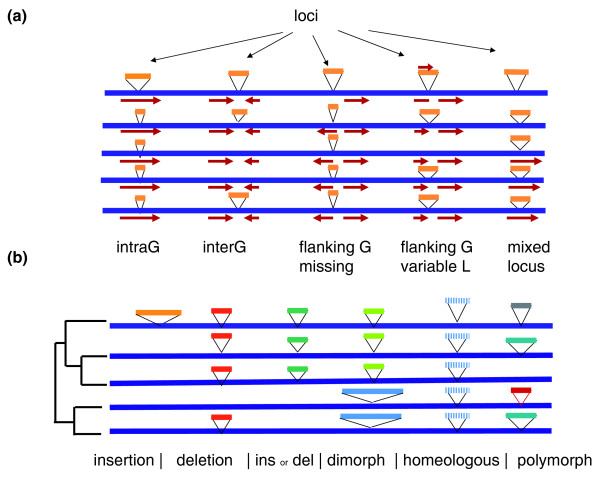
**Rationale for the alignment analyses**. The five horizontal blue lines represent the backbone DNA, and the triangles represent the VSs interrupting the backbone. All the VSs present at a given position of the alignment constitute a locus. **(a)** The five categories of VS positions relative to genes. Red arrows below the backbone blue lines represent genes. IntraG, intragenic; interG, intergenic; G, gene; L, length. **(b)** Loci history. VSs are colored according to DNA content. Identical color indicates identical content. Detection of insertions, deletions, ancient insertion or deletion event (ins or del), dimorph, homeologous and polymorph loci are as detailed in the text.

**Figure 3:**
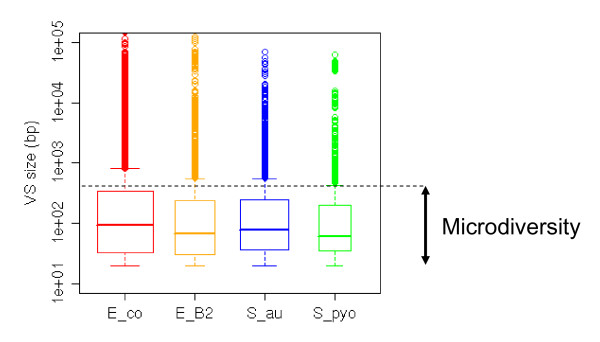
**Size distribution of the variable segments produced in the four alignments (box plots)**. Each box shows the median value (middle lane), first and third quartiles (lower and upper lanes) of the size distribution. Values laying more than 1.5 times the inter-quartile value away from the bulk of all values are shown individually as dots. The width of each box is proportional to the number of VSs analyzed per alignment. On the right side, VSs shorter than 500 bp are designated by microdiversity. Abcissa: E_co, *E. coli*; E_B2, *E. coli *B2 phylogenetic group; S_au, *S. aureus*; S_pyo, *S. pyogenes*.

We will hereafter refer to 'locus' as the position of an alignment where the backbone is interrupted by a VS in at least one strain (Figure 2). The number of loci in a given alignment varied from 344 to 1,037 depending on the species studied (Table 1). The VS size distribution in all four alignments is represented as a box-plot in Figure 3, and whole distributions are shown in Additional file 3. A remarkable feature of all the alignments was that most of the segments were small: the VSs had a median size of 60 to 90 bp (Table 1), and at least 75% of all VSs were smaller than 500 bp (Figure 3). Loci where all VSs were less than 500 bp long were also abundant (62 to 73% of all loci; Table 1), and will be designated hereafter as microdiversity loci. To test whether microdiversity was still present when more genomes are aligned, alignments of *E. coli*, *S. aureus *and *S. pyogenes *using 25, 11 and 12 genomes, respectively, were realized (Table 2). Overall, the number of loci increased by 50% for *E. coli*, 26% for *S. aureus*, and 65% for *S. pyogenes*. Again, microdiversity loci represented 55 to 78% of all loci. We conclude that the most abundant type of genomic diversity is microdiversity, irrespective of the number of genomes included in the alignment.

**Table 2:** Microdiversity loci, including homeologous and dimorphic loci, are dominant categories irrespective of the number of genomes aligned

	*E. coli*		*S. aureus*		*S. pyogenes*	
Number of genomes aligned	5	25	5	11	5	12
Total number of loci	1,037	1,553	768	970	344	570
Number of microdiversity loci (M)	640 (62%)^a^	852 (55%)	556 (72%)	715 (74%)	250 (73%)	385 (67%)
Insertions/M	7.03%	3.99%	3.6%	1.12%	4.8%	5.71%
Deletions/M	4.22%	4.69%	4.68%	4.48%	12.4%	10.91%
Insertions or deletions/M	3.59%	0.47%	3.24%	2.66%	0.8%	0%
Dimorphs/M	37.97%	23.71%	42.63%	52.03%	31.6%	22.34%
Homeologous/M	30.31%	45.89%	22.84%	23.5%	19.6%	27.53%
Polymorphs/M	16.88%	21.24%	23.02%	16.22%	30.8%	33.51%

^a^Percentage of total loci.

Given the abundance of annotated data available for *E. coli *in databases, we selected this species to perform a mapping of the VSs to available annotations such as bacteriophages, genomic islands, clustered, regularly interspaced short palindromic repeats (CRISPRs), ISs, and repeated elements such as minisatellites and PUs (see Materials and methods for data collection). If more than 50% of the length of a VS corresponded to an annotated region, the VS was labeled as such. All VS labels were then stored collectively at the locus level. The number of loci containing each type of annotation is reported (Table 3). Only 35% of the 1,037 loci of the *E. coli *alignment, and 47% of the B2 subgroup loci, corresponded to one of the elements described above. Therefore, the major proportion of the loci does not originate from readily identifiable events. In particular, the microdiversity loci accounted for 63 to 72% of the category 'Other'. The DNA content of the *E. coli *loci not belonging to known categories was compared by Blast to the Non-Redundant database (see Materials and methods). The largest category comprised segments that matched with other *E. coli *strains (65 to 86% of the cumulated DNA length of all VSs tested in a given genome). This suggests that most of the VSs belong to a shared pool of *E. coli *sequences, the so-called *E. coli *pan-genome. The next largest category included segments that did not have any match in the database (13 to 34%). DNA segments matching to other species or environmental samples were essentially absent. In conclusion, most of the variable loci are microdiversity loci, and to the best of our knowledge for *E. coli*, they do not correspond to known elements, although most contain pan-genomic DNA.

**Table 3:** Number of loci in *E. coli *alignments corresponding to known elements

	*E. coli*				*E. coli *B2			
		
	All loci		Microdiversity loci		All loci		Microdiversity loci	
				
	n	Percent	n	Percent	n	Percent	n	Percent
Total	1,037	100	640	100	539	100	370	100
Bacteriophages	27	3	0	0	35	6	12	3
CRISPR	3	0.3	1	0.1	3	2	1	0.2
Genomic islands	127	12	61	10	103	19	64	17
Insertion sequences	55	5	2	0.3	48	9	8	2
Palindromic units	129	12	105	16	44	8	37	10
Minisatellites	18	2	12	2	17	3	15	4
Other	678	65	459	72	289	53	233	63

CRISPR, clustered, regularly interspaced short palindromic repeat.

### Identification of the microdiversity regions possibly affecting genes

The remaining part of this analysis focuses on the microdiversity loci that correspond to largely unknown aspects of genome diversity. We chose to focus on the five-genome alignments because more information was available for these. We asked how microdiversity regions were located respective to genes. A microdiversity locus was designated as an 'intragenic locus' if all VSs of the locus were located inside a gene, without perturbing its reading frame, and as an 'intergenic locus' if all VS boundaries were located outside genes (Figure 2a, first two examples). We also considered the cases where insertion of a VS interrupts a gene in at least one strain of the alignment (such as with IS insertions), and called this category 'flanking gene missing' (Figure 2a, third case). Addition of DNA can also sometimes provoke an in-frame fusion, resulting in a locus where VSs have 'flanking genes of variable length'. Finally, we placed the remaining loci in the 'mixed locus' category (it can correspond, for instance, to loci where some VSs of a given locus are intragenic and others intergenic).

**Figure 4:**
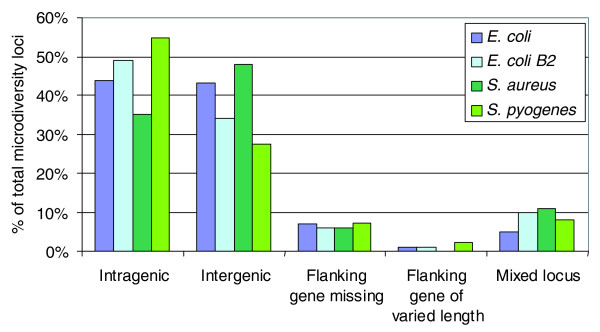
**Location of the variable segments relative to genes in the four alignments**. The proportion of each category is given as percentages of total loci present in each alignment.

**Figure 5:**
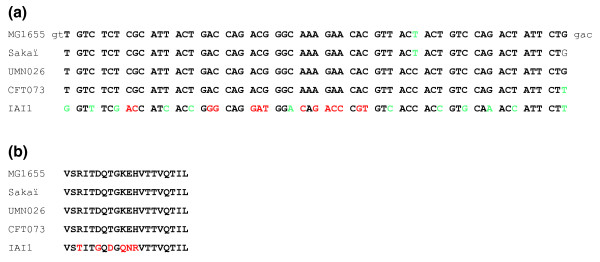
**The 61 bp-long variable segment of the *manZ *gene**. **(a) **DNA sequence. Bold capitals delineate the VS. Non-synonymous mutations are shown in red, synonymous in green. **(b) **Protein sequence. Amino acid changes are shown in red. This locus is intragenic and dimorphic.

Thirty-five to 55% of the microdiversity loci were intragenic (Figure 4), and did not perturb the reading frame of the gene (for example, see the nucleotide sequence of a 61-bp microdiversity locus present in the *manZ *gene; Figure 5). The number of genes affected by microdiversity, that is, harboring a VS in at least one genome, was then calculated. Depending on the genome and the alignment, their proportion ranged from 3 to 6% of all genes. Some genes contained more than one VS. Remarkably, some *S. aureus *genes harbor up to seven in-frame VSs. These *S. aureus *VS-rich genes encode surface proteins such as the fibrinogen binding protein SdrE, or clumping factor ClfB. The most VS-rich gene of *E. coli *and B2 subgroup alignments is *ftsK *(four and three VSs, respectively), encoding a membrane protein important for chromosome segregation. In most cases (75 to 92% of intragenic loci), the amino acid sequence of the protein was modified by the presence of the VS. Complete lists of these genes are given in Additional files 4, 5, 6 and 7, with a break-down according to functional categories for *E. coli *genes in Additional file 8. Genes encoding membrane proteins were significantly enriched among the population of genes with microdiversity loci in the *E. coli *and B2 lists (Additional file 8). These results suggest that besides point mutations, genes also evolve by more abrupt, 'block modifications' of gene fragments (see Discussion).

Intergenic loci represented 23 to 48% of all loci (Figure 4). In *E. coli*, some of them corresponded to PU/repetitive elements (93 of 276 for the global *E. coli *alignment, and 32 of 127 for the *E. coli *B2 subgroup alignment). In the *S. aureus *alignment, the intergenic loci were the most abundant, representing 48% of all variable loci. Some of them likely correspond to *Staphylococcus *repetitive elements [56] that are intergenic, or to staphylococcal interspersed repeats units [57]. An analysis was performed on loci where VSs were located less than 500 bp upstream of an ORF (Additional files 9, 10, 11, and 12), and a break-down in functional categories was effected for the *E. coli *genes (Additional file 13). The proportion of genes preceded by a VS ranged from 1 to 9% of all genes. Non-coding RNA (corresponding to tRNA, rRNA and small non-coding RNA) were significantly enriched among the genes preceded by a VS (Additional file 13). Note that these RNA were not target sites for genomic island integration, which preferentially integrate downstream from tRNAs. They often corresponded to variations in runs of tRNA genes, or in tRNA interspersed between rRNA genes. Apart from this special category, we suspect that the presence of VSs upstream of genes may affect regulation, and hence contribute to strain diversity.

The mixed loci (5 to 10% of all loci) correspond generally to cases where the VSs are either intragenic or intergenic. This suggests mutagenic insertion of a DNA sequence inside a gene, leading to its pseudogenization in the strains where the locus is intergenic. Some additional cases of pseudogenization may be detected in loci with a flanking gene missing (5 to 7% of all loci; Figure 4), if the gene loss is due to the introduction of the VS.

### Some 10% of the VSs are flanked by direct repeats in the microdiversity loci

Recombination between directly oriented repeats placed at the base of the VS may explain one mechanism of variability: in some strains, a deletion may have occurred between repeats, thereby generating a new locus in the alignment. The percentage of VSs flanked by repeats varied between 10 and 18%, with the highest frequency occurrence in *S. aureus *(Table 4, first part). The vast majority (66 to 94%) of repeat sequences were less than 30 bp in size.

**Table 4:** Characteristics of microdiversity loci flanked by repeats

	*E. coli*	*E. coli *B2	*S. aureus*	*S. pyogenes*
VS analysis				
VS flanked by repeats/all VS	10%	14%	18%	12%
Repeats less than 30 bp/all VS with repeats	74%	66%	82%	94%
				
Loci analysis				
Total number of loci	640	370	556	250
% of loci with VSs flanked by repeats (r-loci)/all loci	21%	22%	32%	23%
% loci with possible deletion/r-loci	51%	66%	16%	42%
% loci with possible deletion/all loci	21%	25%	22%	20%

If repeats are responsible for instability, one would expect to find genomes in which the VS is deleted. Loci at which at least one of the VSs was flanked by repeats were designated 'r-loci' (Table 4, second part). Among these r-loci, the proportion of those where at least one genome had an empty VS at the locus (empty VS means the VS is absent or less than 20 bp long) could be calculated (Table 4, last lines). For the *E. coli *and *S. pyogenes *alignments, this proportion was 42 to 66%, which is significantly higher than expected (*P *<< 0.01). For *S. aureus*, the proportion of r-loci with apparent deletions was only 16%, which is even less than the overall proportion of loci with apparent deletions (22%). We conclude that for the r-loci, variability may be explained in part by recombination between these repeats; these events appear to be more frequent in *E. coli *and *S. pyogenes *than in *S. aureus*. Overall, up to one-fifth of the microdiversity between genomes may be due to recombination between short repeats flanking some of the VSs.

### Global prediction of loci history reveals two important categories of events: dimorphic loci, and highly divergent loci

A global analysis was carried out to investigate the possible history of loci and assess the contribution of deletions, insertions, and more complex situations. This implied the analysis of VS content, placed within a phylogenetic context. Our approach consisted first in assigning an 'occupancy' value to all loci. It corresponds, for a given locus, to the number of genomes that 'occupy' the locus, that is, where the VS is not empty. We observed that 75 to 80% of loci had maximal occupancy, that is, occupancy 5 (Additional file 14).

We then made use of locus occupancy, strain phylogeny and VS content to predict some simple situations, using the parsimony principle (Figure 2b): loci of occupancy 1 with VSs on a short branch were predicted to be 'recent insertions', while loci of occupancy 4 with identical VS content and the longer branch occupied were predicted as 'recent deletions'. Using a similar method, loci of occupancy 2 or 3 with VSs of identical content present on the same sub-tree, were predicted as 'ancestral insertions or ancestral deletions'. Among the loci of maximal occupancy, two situations were singled out: loci with only two kinds of VS segregating on subtrees, which were named 'dimorphs'; and loci where all VSs turned out to be of nearly identical content, which were named 'homeologs'. These loci may indicate places where DNA diverges more rapidly than elsewhere on the genome, and they were therefore kept in the 'VS pool'. The last category of 'polymorphs' included all other loci.

**Figure 6:**
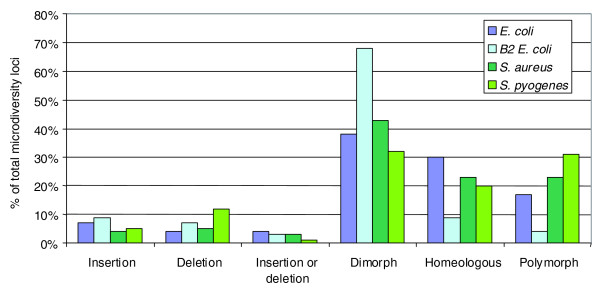
**Prediction of locus histories in the four alignments**. The proportion of each category is given as percentages of total loci present in each alignment.

Results showing the proportions of loci encountered in each category are reported in Figure 6. Surprisingly, the 'dimorphs', in which a given locus contains exactly two different kinds of segment, was the most abundant category. Dimorphic loci can be explained by the presence of a DNA insertion hot spot or by the replacement of an 'ancestral' sequence by a new segment. If such is the case, it should be possible to match one of the two VSs of the locus with a genome segment of a closely related species. A Blast analysis was conducted for the *E. coli *and B2 phylogenetic group alignments on all dimorphic loci, using *Escherichia fergusonii *as an out-group [53]. In 55% of *E. coli *loci, and 36% of the B2 group loci, a matching segment with *E. fergusonii *was found (76% identity on 90% of its length). This argues for the existence of a segment replacement in a fraction of the dimorphs. A comparable matching could not be performed for the two other species due to the absence of a sufficiently proximal genome out-group.

Homeologous loci represented 9 to 30% of the total loci (see Figure 5 for an example of such an homeologous locus). Interestingly, the longer the maximal MUMi genomic distance among the strains being compared, the higher the proportion of divergent loci among the total VSs. This may suggest that the yield of divergent loci reflects the evolutionary time elapsed from the time that the species diverged. The homeologous loci were significantly enriched among the intragenic loci for two alignments: *E. coli *(53% of intragenic loci are homeologous, compared to 30% homeologous loci overall, *P *<< 0.01), and *S. aureus *(33% compared to 23%, *P *= 0.017). This was not the case, however, for the B2 *E. coli *alignment (14% compared to 9%, *P *= 0.08), or the *S. pyogenes *alignment, where 23% of intragenic loci are homeologous, compared to 20% overall.

The polymorphic loci included 4 to 31% of all microdiversity loci, and may correspond to recombination hotspots, which remain to be studied in detail.

We then proceeded to test whether the two most important categories identified with the five-genome alignments, namely dimorphic and homeologous loci, were conserved when more genomes were included in the alignment. This proved to be the case (Table 2). For the *E. coli *and the *S. pyogenes *alignments, the homeologous loci even became preponderant relative to the dimorphic loci.

In conclusion, microdiversity loci correspond mostly to cases of segment replacement, recombination hot spots, or to homeologous DNA that diverged faster relative to the backbone DNA. Cases of simple deletion or insertions were scarce, proportionally.

## Discussion

### Microdiversity constitutes a major type of variability between bacterial genomes within a species

The main outcome of this study is the discovery of a major type of bacterial genome diversity at the species level, made of variable short segments between 20 and 500 bp long. In the five-genome alignments, these VSs represent some 63 to 72% of all possible variable regions detected by whole genome alignments. They remain very abundant (50 to 72% of all loci) when a maximal number of genomes are included in the alignments (Table 2). The presence of such small diversity had been reported earlier for *E. coli *[4,58], and its general importance is presently emerging in various comparative genomic studies, both in eukaryotes [59] and prokaryotes [60], where it is often reported as indels. However, the term indel is imprecise with respect to the size of segments involved (it can be used for 1- to 10-bp insertions or deletions up to the insertion or deletion of genomic islands). It is also misleading in terms of the underlying mechanism because it suggests that an insertion or a deletion occurred. Our work shows that more than 80% of the microdiversity loci are due to neither insertion nor deletion. The term indel was therefore replaced in this study by the more neutral term of microdiversity. If such microdiversity were found essentially outside genes, it might be considered as recombination scars, with little evolutionary importance. However, among the five-genome alignments, 35 to 55% of microdiversity regions lie within ORFs and 16 to 33% of VSs are immediately upstream of ORFs. They should therefore contribute greatly to strain diversity within a species, either by affecting protein domains or by changing gene expression.

Among the *E. coli *genes harboring microdiversity, those encoding membrane and surface proteins are significantly enriched in VSs. This is in keeping with the notion that bacteria adapt to their varying and challenging environments by modifying their surface proteins, as already documented [61]. A comparative genome analysis detected 23 genes that are under positive selection in *E. coli *[62]. The present study identifies six of them (*fhuA*, *ompA*, *ompC*, *ompF*, *lamB *and *ubiF*) as harboring microdiversity. Moreover, for five of the six proteins where the structure is known, the Peterson analysis revealed that all mutations were concentrated on one or a few loops of the protein [62]; this feature allowed us to detect them in our screen, as scattered mutations would have gone undetected. Recently, using a more sensitive approach, 290 core genes of *E. coli *were detected as under short-term positive selection [63]. However, only four of them (*narH, fes*, *cstA *and *yphH*) corresponded to the 192 genes we report here as harboring microdiversity. Therefore, at least 10 of the 192 genes harboring microdiversity may be under positive selection. Interestingly, microdiversity regions have been found in orthologous proteins compared broadly across bacterial and yeast species and found to be more numerous in essential proteins, which suggests a functional role for these flexible regions [60].

### Illegitimate recombination may explain a large fraction of the VSs

One aim of this study was to elucidate the mechanisms underlying DNA recombination in microbial genomes. To this end, we focused on *E. coli*, the best studied bacterial species at the molecular level for recombination. More than half of the VS loci could not be explained by site-specific recombination, nor by transposition, nor by the hypothetical mechanism invoked for very short dispersed elements similar to PUs [29] (Table 2). We speculate that homologous or illegitimate recombination may explain these loci: in the three species, analysis of the five-genome alignments have shown that 10 to 18% of the VSs are flanked by repeats at least 5 bp long, which might account for part of the variability, especially as a deletion was often found associated with such loci (Table 4). However, as most repeats were of a size below 30 bp, the reported threshold for RecA-dependent homologous recombination in *E. coli *[64], it is likely that VSs are generated by replication slippage between the repeats, a mechanism also called short-homology-dependent illegitimate recombination [65]. Although not as proportionally abundant as events detected in a previous, more limited study [50], the present analysis implicates short-homology-mediated deletion events as one significant cause of genome variability.

This conclusion on the importance of illegitimate recombination with regards to the VSs should not yield to the notion that homologous recombination is unimportant in bacterial genomes. Rather, homologous recombination relies on the detection of subtle tracts of 3 to 4% diverged sequences, which are not taken into account in our VS analysis. These sequences are part of the backbone, and studies on backbone DNA detecting blocks of mutations moving together across strains have shown, to the contrary, that homologous recombination plays a great role in bacteria. In *E. coli*, the average size of these blocks was estimated to be 500 bp in a first study on four genomes [66], and more recently re-estimated to to 50 bp based on a 20-genome comparison [53]. It has also been demonstrated that genomic islands, once integrated into a genome (by site-specific recombination most likely), diffuse in a population by homologous recombination between the sequences flanking the island [9].

**Figure 7:**
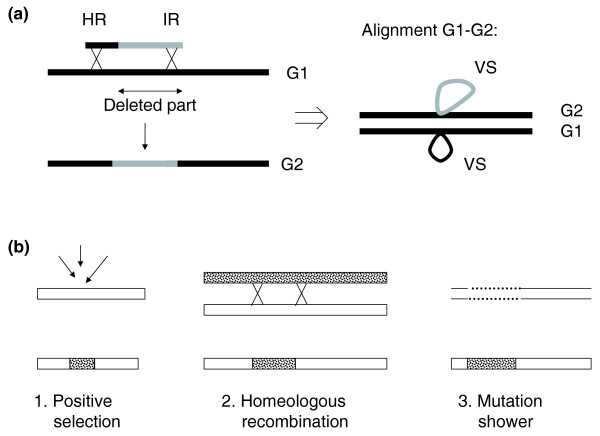
**Possible mechanisms explaining dimorphic and homeologous loci**. **(a)** Dimorphic loci. Incoming DNA (the shorter, black and grey molecule above) may recombine by illegitimate recombination assisted by homology with the resident bacterial chromosome G1. HR, homologous recombination; IR, illegitimate recombination; G1 and G2, genomes 1 and 2; VS, variable segment. **(b)** Three possible scenarios to explain the origin of microdiversity at homeologous loci in bacterial genomes (see text for details).

Dimorphic loci, which contain exactly two different segments at a given site, represent 38 to 68% of all loci in the five-genome alignments (Figure 6), and 22 to 52% of all microdiversity loci in the maximal alignments (Table 2). In the case of the *E. coli *five-genome alignment, we found that in about half the cases, one of the two segments was present in *E. fergusonii*. This suggests that the ancestral segment was replaced at some point by another segment. A process called 'illegitimate recombination assisted by homology' can produce such a situation [67-69]. If the new incoming DNA segment is flanked by a segment homologous to the recipient chromosome, RecA may initiate homologous recombination on part of the molecule, followed by 'illegitimate' actors that complete the DNA integration at the other extremity (Figure 7a). Such a process is described in *Streptococcus pneumoniae*, *Acinetobacter baylii *and *Pseudomonas stutzeri*, three naturally competent species, and was found to be 10^2^- to 10^5^-fold more efficient than strict illegitimate recombination [67-69]. Whether such a process could occur in *E. coli*, for instance during DNA conjugation, is presently under study. Alternatively, dimorphic (as well as polymorphic) loci may also correspond to fragile sites of the chromosome, which are hot spots of illegitimate recombination.

Although illegitimate recombination occurs at low frequency, our analysis of VSs suggests that it nevertheless is responsible for a large proportion of the genomic diversity: taking all loci differing from known events for *E. coli*, and labeled "Other" in Table 3, and removing the category of homeologous loci (Figure 6) we estimate that it is responsible for 41% (*E. coli *five-genome alignment) to 56% (*E. coli *B2 alignment) of microdiversity loci.

### What mechanism generates homeologous DNA microdiversity?

A particular class of loci comprises those containing homeologous sequences. For *E. coli*, *S. aureus *and *S. pyogenes*, they represent 20 to 30% of loci in the five-genome alignments, and even more (20 to 46%) in the maximal genome alignments (Table 2). They are less abundant, however, in the alignment of B2 genomes (9%). Interestingly, we found that among the five-genome alignments, homeologous loci were significantly enriched among intragenic loci (50 to 78% of the divergent loci are intragenic). The question arises as to how such blocks of microdiversity could be generated. Three scenarios are considered: positive selection, homeologous recombination and mutation showers (Figure 7b).

#### Positive selection

A given protein domain may be under positive selection, so that non-synonymous mutations accumulate in a limited region of the corresponding gene, while conservation of the rest of the protein is selected by physical constraints (for example, membrane-spanning domains), such that non-synonymous mutations are counter-selected. In contrast, synonymous mutations are expected in equal density inside and outside the microdiversity block. However, we did not observe this pattern (synonymous mutations were also enriched in the homeologous loci), and therefore tend to exclude this hypothesis.

#### Homeologous recombination between diverged DNA segments

Given our similarity threshold, recombination should have taken place between at least 24% diverged sequences. In *E. coli*, RecA seems inefficient on 22% diverged sequences [70], and *B. subtilis *RecA is apparently inhibited by 7% divergence [71]. However, phage recombinases may be more efficient on highly diverged DNA [70]. Moreover, it is suspected that, in nature, bacteria alternate between a mutator and non-mutator state, via the inactivation/activation of the *mutS *or *mutL *genes, and during the mutator period, homeologous recombination should increase [72].

#### Mutation showers

High mutation densities are sometimes observed both in eukaryotes [73]and prokaryotes [74], and it is suggested that local exposure to a mutagenic agent, or a long state as single strand DNA may result in such mutation showers [75].

## Conclusions

We report here an attempt to examine systematically genome variability at the DNA level in several bacterial species. We have shown that at the species level, the main kind of genomic variability is 'microdiversity'. It consists of small blocks (20 to 500 bp in length) of DNA, often present within or upstream of genes and contributing to the genome diversity. This notion raises the question of the mechanisms that may generate such diversity, and opens challenging new questions at both the molecular and bacterial evolution level.

## Materials and methods

### Genomes

All publicly available complete sequences and annotations were downloaded from the Genome Reviews database [76]. *S. aureus *genomes: Mu50 [GenBank: BA000017], MW2 [GenBank:BA000033], COL [GenBank:CP000046], RF122 [GenBank:AJ938182], MRSA252 [GenBank:BX571856], N315 [GenBank:BA000018], JH1 [GenBank:CP000736], MSSA476 [GenBank:BX571857], NCTC8325 [GenBank:CP000253], Newman [GenBank:AP009351], USA300 [GenBank:CP000255]. *S. pyogenes *genomes: M1 GAS, also known as SF370 [GenBank:AE004092], GAS315 [GenBank:NC004070], GAS8232 [GenBank:NC003485], GAS2096 [GenBank:NC008023], GAS10270 [GenBank:NC008022], GAS9429 [GenBank:CP000259], GAS10750 [GenBank:CP000262], NZ131 [GenBank:CP000829], GAS5005 [GenBank:CP000017], GAS6180 [GenBank:CP000056], GAS10394 [GenBank:CP000003], Manfredo [GenBank:AM295007]. *E. coli *genomes: K-12 MG1655 [GenBank:U00096], O157:H7 Sakai [GenBank:BA000007], B2 phylogenetic group, strain CFT073 [GenBank:AE014075], B2 group, strain UTI89 [GenBank:CP000243], B2 group, strain APECO1 [GenBank:CP000468], B2 phylogenetic group, strain 536 [GenBank:CP000247], B2 phylogenetic group, strain S88 [GenBank:CU928161], W3110 [GenBank:AP009048], DH10B [GenBank:CP000948], BW2952 [GenBank:CP001396], REL606 [GenBank:CP000819], BL21 [GenBank:AM946981], HS [GenBank:CP000802], Crooks [GenBank:CP000946], 55989 [GenBank:CU928145], E24377A [GenBank:CP000800], SE11 [GenBank:AP009240], EDL933 [GenBank:AE005174], TW14359 [GenBank:CP001368], 4115 [GenBank:CP001164], SMS3-5, named SECEC here [GenBank:CP000970], IAI39 [GenBank:CU928164], B2 phylogenetic group, E2348-69 [GenBank:FM180568]. All *E. coli *genome annotations were downloaded from the Genoscope ColiScope project [77], and their annotations were homogenized using the MaGe annotation platform [78].

### Alignment strategies

A first set of alignments involving few and collinear genomes were computed using the MGA software [2]. Genomes were selected so as to be representative of the species under study. For this, a genomic distance based on maximal unique matches (MUM) was calculated for all possible genome pairs [54], and neighbor-joining trees were built so as to choose the appropriate genomes. When several closely related genomes were available, the second criterion used was genome collinearity, as determined by Mummer plots [79]. MGA alignment parameters were fine-tuned as described [4]. Briefly, in a first step, detection of anchors composed of maximal exact matches of minimal length 50 bp common to all genomes was carried out. A subset of collinear anchors was then selected by a chaining algorithm. Next, these two steps were repeated in each interval framed by the chosen anchors, using a lower minimal length value of 20 bp for the maximal exact matches. The remaining gaps of the alignment, if shorter in length than 3,000 bp, were treated with ClustalW.

MGA alignment outputs are stored in the MOSAIC database after a post-treatment step on the raw ClustalW results. This step is needed to define, among the ClustalW output files, those in which the alignment reflects common ancestry from those where different pieces of DNA are forced into an alignment. As described earlier [4], post-treatment parameters were chosen so as to classify as VSs all segments of a given locus, if at least two of them share less than 76% identity on 100% of the aligned length, or if a gap larger than 20 bp is found in the alignment. This allowed a high sensitivity with respect to VS size, but also some flexibility with respect to overall DNA divergence. The choice of the 76% threshold for DNA identity is described in Additional file 2. The 20-bp gap size was chosen as corresponding, at the protein level, to a small secondary structure of at least six amino acids. The minimal VS size was set to 20 bp. We compared the results obtained when the minimal VS size was increased from 20 to 42 bp for a three-strain *E. coli *and a six-strain *S. aureus *alignment (alignments computed in the preparatory phase of this analysis). This resulted in a 26% decrease in the global number of loci. This indicated that an important proportion of VSs belongs to microdiversity loci, and justified our choice to maintain the minimal VS size as 20 bp, so as to be more sensitive to the microdiversity loci that may contribute to strain diversity.

A second set of alignments were computed so as to include a maximal number of genomes for the *E. coli*, *S. aureus*, and *S. pyogenes *species, using MAUVE version 1.2.3 for *S. aureus *and *S. pyogenes *[1], and progressive MAUVE version 2.1.3 for *E. coli*, instead of MGA for the first step. The same MOSAIC post-treatment step as described above was then applied [5]. Compared to MGA, the MAUVE software offers the advantages of dealing with large rearrangements, and the possibility to treat high numbers of genomes. This comes, however, at the price of slightly less precise backbone/VS boundaries, as we observed when comparing output from MGA versus MAUVE version 1.2.3 for an *E. coli *MG1655-Sakai alignment. Analyses requiring precise VS boundaries, such as repeat detection and positions of VSs relative to genes, were thus restricted to the MGA alignments. The phylogenetic trees corresponding to the strains used for the alignments are shown in Additional file 1.

### Collection of additional annotations for the E. coli genomes

#### Bacteriophages

Phage coordinates of strains MG1655 and Sakai were downloaded from the Sakai genome project web page [80]. For the CFT073, UTI89 and 536 genomes, the Prophinder tool [19] and web access were used [81].

#### CRISPR sequences

Positions of the CRISPR sequences were retrieved from the CRISPR database of G Vergnaud's laboratory [82].

#### Genomic islands

Ou *et al*. [16] described a systematic means to detect genomic islands. Coordinates were downloaded from the supplementary data provided by them for MG1655, Sakai and CFT073 genomes. For the other genomes, an approach similar to that of Ou *et al*. based on synteny break points was used. Briefly, blocks of genes at least 5 kb long and not following the local synteny are analyzed for exceptional GC content or interpolated variable order motif (IVOM) value [83], presence of flanking tRNA genes, and presence of integrase-like genes. All blocks meeting at least one of the criteria were considered as regions of genomic plasticity, a denomination that does not make any assumption about the evolutionary origin or genetic basis of these variable chromosomal segments. The regions corresponding to bacteriophages and CRISPRs as defined above were then removed, and counted separately.

#### Insertion sequences

For all genomes but S88, UMN026 and IAI1, IS coordinates were taken from the ASAP site [84]. For the three remaining genomes, ISs were detected by the presence of transposase genes.

#### Palindromic units/repetitive sequence elements

PUs, also called repetitive sequences, have been described for *E. coli *[30]. Their coordinates on MG1655 were calculated starting from the Bachelier web page [85], and converting the coordinates so that they match with the current version of the MG1655 genome. Detection of putative PUs on the other *E. coli *genomes was performed as follows. PUs being palindromic, the presence of half a PU was searched using fuzznuc (EMBOSS package), with the following pattern ' [ACG] [AT] [TC]GCC [GT]GATGCGN(3,9)CG [CT](0,1)CTTATC [CA] GGCCTAC [AG]' allowing for a maximum of four mismatches. PUs are often associated in pairs, which form bacterial interspersed mosaic elements. PUs separated by less than 100 bp were therefore associated in a unique mosaic element. Application of this pattern to the MG1655 complete genome allows detection of 80% of the 266 PUs or mosaics described in [85].

#### Minisatellites

Genomes were searched for tandemly repeated sequences on the minisatellite database of G Vergnaud's laboratory [86]. Parameters used were repeat motifs at least 20 bp long, repeated at least twice, such that identity between repeats is at least 90%. Among the minisatellites, a majority corresponded to PU elements that were scored separately (see above), so that only the remaining, non-PU minisatellites were reported in this category.

#### Source of other *E. coli *variable segment

For all *E. coli *VSs that did not correspond to the above mentioned annotations, an estimation of their content was carried out using Blast against the EMBL Non-Redundant database, and the result was considered positive if at least 90% identity over at least 90% of the length was obtained. Results were parsed using the following categories: DNA segment present in at least one other *E. coli *strain (except very close kin such as EDL933, which is clonally related to the Sakai strain, or W3110, related to MG1655); DNA segment present in another bacterial species or a non-cultivable sample; no match in the Non-Redundant database.

### Variable segment analysis

#### Data preparation

Coordinates of the VSs for all four alignments were downloaded from the MOSAIC web site [55]. A script written in Python allowed us to analyze the VSs, in which the central object was the 'locus' class, composed of all VSs belonging to the same locus. Boundaries of some of the VSs as generated by the aligner were sometimes inexact, in the sense that the DNA content of the boundary (usually not more than 20 to 100 bp) was more than 90% identical in all VSs. A pre-treatment of the VS arrays was therefore performed to trim such boundaries (and sometimes remove a VS when its size shrank below 20 bp). As a result, some of the VSs described in the MOSAIC interface are slightly larger than those considered in this study.

#### Inspection of variable segment boundaries relative to backbone genes

For all VSs, a right and left neighboring gene on the backbone was assigned (the neighbor gene either overlapped the VS or was the first gene next to it). The position of all VSs of a given locus, relative to these genes, was then analyzed. If all VSs were inside genes, meaning that the ORF of the genes in all genomes was not interrupted by any of the VSs of the locus, the locus was labeled intragenic. If all VSs were between two genes that did not overlap with the VS boundaries, the locus was labeled intergenic. A flanking gene on the backbone was considered as missing if, among all VSs of the locus, the distance between one VS boundary and its neighboring gene distal extremity varied by more than 500 bp (that is, the approximate size of a small gene). When the flanking gene overlapped with a VS boundary, the gene portion lying inside the VS was compared with all VSs: if this portion varied by more than 50 bp (approximately 16 amino acids), it was considered that the locus modified the length of the flanking gene. If the neighbor genes overlapped the VS by less than these 50 bp, the overlapping was considered negligible and the locus was considered as intergenic.

#### Detection of repeats flanking variable segments

For all VSs, a DNA fragment encompassing the VS and 500 bp flanking each side was extracted. Repeat detection was done with the Vmatch software [87], using a three step procedure. First, VS boundaries were scanned for the presence of repeats of length = 11 bp, allowing 10% divergence between the repeats, and a misplacement of the repeat of 10 bp around the position of the VS boundary. If no repeat was found, a second search of repeats of length >10 bp with a Hamming distance of 1 was carried out. A final scan was done in case of repeat detection failure, for exact repeats ≥ 5 bp (this value was chosen based on an example of a known, accurate deletion of genes *yafN *and *yafO *that occurred between a 5-bp repeat in the CFT073 strain of *E. coli*), allowing no misplacement relative to the VS boundary (otherwise, the probability to find such repeats at random is too high). This last step was found to double the number of VSs flanked by repeats.

#### Detection of variable segments with similar DNA content

To determine which VSs of a locus had similar content, pairwise alignments on VSs having similar lengths (± 10%) were performed using 'stretcher' (EMBOSS suite). A similar content was attributed if more than 76% identity was found over at least 90% of the smaller VS length. A final step controlled that all relationships within the locus were transitive.

## Abbreviations

bp: base pair; CRISPR: clustered: regularly interspaced short palindromic repeat; IS: insertion sequence; ORF: open reading frame; PU: palindromic unit; VS: variable segment.

## Authors' contributions

FT performed the analysis of maximal genome alignments, MAP conceived the work, performed it, and wrote the manuscript. ED, CM and VB were responsible for the complete sequencing and annotation of seven strains of *Escherichia *(ColiScope project), and made their data available prior to publication. MEK contributed to the work and ED and MEK contributed to the manuscript.

## Supplementary Material

Additional file 1 Neighbor joining trees based on genomic MUMi distances of the strains selected for the maximal genomes alignments. Click here for file

Additional file 2 Choice of the maximum divergence level for inclusion of ClustalW aligned sequences into the backbone. Click here for file

Additional file 3 Distribution of the VS sizes in the five-genome alignments. Click here for file

Additional file 4 List of genes containing microdiversity loci in the *E. coli *five-genome alignments. Click here for file

Additional file 5 List of genes containing microdiversity loci in the *E. coli *B2 five-genome alignments. Click here for file

Additional file 6 List of genes containing microdiversity loci in the *S. aureus *five-genome alignments. Click here for file

Additional file 7 List of genes containing microdiversity loci in the *S. pyogenes *five-genome alignments. Click here for file

Additional file 8 Distribution of the *E. coli *genes containing microdiversity loci in functional categories. Click here for file

Additional file 9 List of genes placed downstream of microdiversity loci in the *E. coli *five-genome alignments. Click here for file

Additional file 10 List of genes placed downstream of microdiversity loci in the *E. coli *B2 five-genome alignments. Click here for file

Additional file 11 List of genes placed downstream of microdiversity loci in the *S. aureus *five-genome alignments. Click here for file

Additional file 12 List of genes placed downstream of microdiversity loci in the *S. pyogenes *five-genome alignments. Click here for file

Additional file 13 Distribution of the *E. coli *genes placed downstream of microdiversity loci in functional categories. Click here for file

Additional file 14 Loci occupancy in the five-genome alignments. Click here for file

## References

[B1] Darling AC Mau B Blattner FR Perna NT Mauve: multiple alignment of conserved genomic sequence with rearrangements. Genome Res 2004 14 1394 1403 10.1101/gr.2289704 15231754 PMC442156

[B2] Hohl M Kurtz S Ohlebusch E Efficient multiple genome alignment. Bioinformatics 2002 18 Suppl 1 S312 320 12169561 10.1093/bioinformatics/18.suppl_1.s312

[B3] Treangen TJ Messeguer X M-GCAT: interactively and efficiently constructing large-scale multiple genome comparison frameworks in closely related species. BMC Bioinformatics 2006 7 433 10.1186/1471-2105-7-433 17022809 PMC1629028

[B4] Chiapello H Bourgait I Sourivong F Heuclin G Gendrault-Jacquemard A Petit MA El Karoui M Systematic determination of the mosaic structure of bacterial genomes: species backbone versus strain-specific loops. BMC Bioinformatics 2005 6 171 10.1186/1471-2105-6-171 16011797 PMC1187871

[B5] Chiapello H Gendrault A Caron C Blum J Petit MA El Karoui M MOSAIC: an online database dedicated to the comparative genomics of bacterial strains at the intra-species level. BMC Bioinformatics 2008 9 498 10.1186/1471-2105-9-498 19038022 PMC2607288

[B6] Hayashi T Makino K Ohnishi M Kurokawa K Ishii K Yokoyama K Han CG Ohtsubo E Nakayama K Murata T Tanaka M Tobe T Iida T Takami H Honda T Sasakawa C Ogasawara N Yasunaga T Kuhara S Shiba T Hattori M Shinagawa H Complete genome sequence of enterohemorrhagic *Escherichia coli *O157:H7 and genomic comparison with a laboratory strain K-12. DNA Res 2001 8 11 22 10.1093/dnares/8.1.11 11258796

[B7] Vernikos GS Parkhill J Resolving the structural features of genomic islands: a machine learning approach. Genome Res 2008 18 331 342 10.1101/gr.7004508 18071028 PMC2203631

[B8] Gill SR Fouts DE Archer GL Mongodin EF Deboy RT Ravel J Paulsen IT Kolonay JF Brinkac L Beanan M Dodson RJ Daugherty SC Madupu R Angiuoli SV Durkin AS Haft DH Vamathevan J Khouri H Utterback T Lee C Dimitrov G Jiang L Qin H Weidman J Tran K Kang K Hance IR Nelson KE Fraser CM Insights on evolution of virulence and resistance from the complete genome analysis of an early methicillin-resistant *Staphylococcus aureus *strain and a biofilm-producing methicillin-resistant *Staphylococcus epidermidis *strain. J Bacteriol 2005 187 2426 2438 10.1128/JB.187.7.2426-2438.2005 15774886 PMC1065214

[B9] Schubert S Darlu P Clermont O Wieser A Magistro G Hoffmann C Weinert K Tenaillon O Matic I Denamur E Role of intraspecies recombination in the spread of pathogenicity islands within the *Escherichia coli *species. PLoS Pathog 2009 5 e1000257 10.1371/journal.ppat.1000257 19132082 PMC2606025

[B10] Chen J Novick RP Phage-mediated intergeneric transfer of toxin genes. Science 2009 323 139 141 10.1126/science.1164783 19119236

[B11] Tormo MA Ferrer MD Maiques E Ubeda C Selva L Lasa I Calvete JJ Novick RP Penades JR *Staphylococcus aureus *pathogenicity island DNA is packaged in particles composed of phage proteins. J Bacteriol 2008 190 2434 2440 10.1128/JB.01349-07 18223072 PMC2293202

[B12] Brochet M Rusniok C Couve E Dramsi S Poyart C Trieu-Cuot P Kunst F Glaser P Shaping a bacterial genome by large chromosomal replacements, the evolutionary history of *Streptococcus agalactiae*. Proc Natl Acad Sci USA 2008 105 15961 15966 10.1073/pnas.0803654105 18832470 PMC2572952

[B13] Burrus V Pavlovic G Decaris B Guedon G Conjugative transposons: the tip of the iceberg. Mol Microbiol 2002 46 601 610 10.1046/j.1365-2958.2002.03191.x 12410819

[B14] Hsiao W Wan I Jones SJ Brinkman FS IslandPath: aiding detection of genomic islands in prokaryotes. Bioinformatics 2003 19 418 420 10.1093/bioinformatics/btg004 12584130

[B15] Mantri Y Williams KP Islander: a database of integrative islands in prokaryotic genomes, the associated integrases and their DNA site specificities. Nucleic Acids Res 2004 32 D55 58 10.1093/nar/gkh059 14681358 PMC308793

[B16] Ou HY Chen LL Lonnen J Chaudhuri RR Thani AB Smith R Garton NJ Hinton J Pallen M Barer MR Rajakumar K A novel strategy for the identification of genomic islands by comparative analysis of the contents and contexts of tRNA sites in closely related bacteria. Nucleic Acids Res 2006 34 e3 10.1093/nar/gnj005 16414954 PMC1326021

[B17] Bose M Barber RD Prophage Finder: a prophage loci prediction tool for prokaryotic genome sequences. In Silico Biol 2006 6 223 227 16922685

[B18] Fouts DE Phage_Finder: automated identification and classification of prophage regions in complete bacterial genome sequences. Nucleic Acids Res 2006 34 5839 5851 10.1093/nar/gkl732 17062630 PMC1635311

[B19] Lima-Mendez G Van Helden J Toussaint A Leplae R Prophinder: a computational tool for prophage prediction in prokaryotic genomes. Bioinformatics 2008 24 863 865 10.1093/bioinformatics/btn043 18238785

[B20] Barrangou R Fremaux C Deveau H Richards M Boyaval P Moineau S Romero DA Horvath P CRISPR provides acquired resistance against viruses in prokaryotes. Science 2007 315 1709 1712 10.1126/science.1138140 17379808

[B21] Marraffini LA Sontheimer EJ CRISPR interference limits horizontal gene transfer in staphylococci by targeting DNA. Science 2008 322 1843 1845 10.1126/science.1165771 19095942 PMC2695655

[B22] Bland C Ramsey TL Sabree F Lowe M Brown K Kyrpides NC Hugenholtz P CRISPR recognition tool (CRT): a tool for automatic detection of clustered regularly interspaced palindromic repeats. BMC Bioinformatics 2007 8 209 10.1186/1471-2105-8-209 17577412 PMC1924867

[B23] Grissa I Vergnaud G Pourcel C The CRISPRdb database and tools to display CRISPRs and to generate dictionaries of spacers and repeats. BMC Bioinformatics 2007 8 172 10.1186/1471-2105-8-172 17521438 PMC1892036

[B24] Siguier P Perochon J Lestrade L Mahillon J Chandler M ISfinder: the reference centre for bacterial insertion sequences. Nucleic Acids Res 2006 34 D32 36 10.1093/nar/gkj014 16381877 PMC1347377

[B25] Wagner A Lewis C Bichsel M A survey of bacterial insertion sequences using IScan. Nucleic Acids Res 2007 35 5284 5293 10.1093/nar/gkm597 17686783 PMC2018620

[B26] Zhou F Olman V Xu Y Insertion Sequences show diverse recent activities in Cyanobacteria and Archaea. BMC Genomics 2008 9 36 10.1186/1471-2164-9-36 18218090 PMC2246112

[B27] Chang CH Chang YC Underwood A Chiou CS Kao CY VNTRDB: a bacterial variable number tandem repeat locus database. Nucleic Acids Res 2007 35 D416 421 10.1093/nar/gkl872 17175529 PMC1781188

[B28] Denoeud F Vergnaud G Identification of polymorphic tandem repeats by direct comparison of genome sequence from different bacterial strains: a web-based resource. BMC Bioinformatics 2004 5 4 10.1186/1471-2105-5-4 14715089 PMC331396

[B29] Elhai J Kato M Cousins S Lindblad P Costa JL Very small mobile repeated elements in cyanobacterial genomes. Genome Res 2008 18 1484 1499 10.1101/gr.074336.107 18599681 PMC2527708

[B30] Gilson E Saurin W Perrin D Bachellier S Hofnung M The BIME family of bacterial highly repetitive sequences. Res Microbiol 1991 142 217 222 10.1016/0923-2508(91)90033-7 1656494

[B31] Valens M Penaud S Rossignol M Cornet F Boccard F Macrodomain organization of the *Escherichia coli *chromosome. EMBO J 2004 23 4330 4341 10.1038/sj.emboj.7600434 15470498 PMC524398

[B32] Anderson RP Roth JR Tandem genetic duplications in phage and bacteria. Annu Rev Microbiol 1977 31 473 505 10.1146/annurev.mi.31.100177.002353 334045

[B33] Lovett ST Drapkin PT Sutera VA Jr Gluckman-Peskind TJ A sister-strand exchange mechanism for recA-independent deletion of repeated DNA sequences in *Escherichia coli*. Genetics 1993 135 631 642 8293969 10.1093/genetics/135.3.631 PMC1205708

[B34] Bruand C Bidnenko V Ehrlich SD Replication mutations differentially enhance RecA-dependent and RecA-independent recombination between tandem repeats in *Bacillus subtilis*. Mol Microbiol 2001 39 1248 1258 10.1111/j.1365-2958.2001.02312.x 11251841

[B35] Marsin S Mathieu A Kortulewski T Guerois R Radicella JP Unveiling novel RecO distant orthologues involved in homologous recombination. PLoS Genet 2008 4 e1000146 10.1371/journal.pgen.1000146 18670631 PMC2475510

[B36] Smith GR Conjugational recombination in *E. coli *: myths and mechanisms. Cell 1991 64 19 27 10.1016/0092-8674(91)90205-D 1986865

[B37] Claverys JP Prudhomme M Mortier-Barriere I Martin B Adaptation to the environment: *Streptococcus pneumoniae*, a paradigm for recombination-mediated genetic plasticity?. Mol Microbiol 2000 35 251 259 10.1046/j.1365-2958.2000.01718.x 10652087

[B38] Chedin F Dervyn E Dervyn R Ehrlich SD Noirot P Frequency of deletion formation decreases exponentially with distance between short direct repeats. Mol Microbiol 1994 12 561 569 10.1111/j.1365-2958.1994.tb01042.x 7934879

[B39] Ikeda H Shimizu H Ukita T Kumagai M A novel assay for illegitimate recombination in *Escherichia coli *: stimulation of lambda bio transducing phage formation by ultra-violet light and its independence from RecA function. Adv Biophys 1995 31 197 208 10.1016/0065-227X(95)99392-3 7625274

[B40] Canceill D Ehrlich SD Copy-choice recombination mediated by DNA polymerase III holoenzyme from *Escherichia coli*. Proc Natl Acad Sci USA 1996 93 6647 6652 10.1073/pnas.93.13.6647 8692872 PMC39080

[B41] Canceill D Viguera E Ehrlich SD Replication slippage of different DNA polymerases is inversely related to their strand displacement efficiency. J Biol Chem 1999 274 27481 27490 10.1074/jbc.274.39.27481 10488082

[B42] Viguera E Canceill D Ehrlich SD Replication slippage involves DNA polymerase pausing and dissociation. EMBO J 2001 20 2587 2595 10.1093/emboj/20.10.2587 11350948 PMC125466

[B43] Vilette D Uzest M Ehrlich SD Michel B DNA transcription and repressor binding affect deletion formation in *Escherichia coli *plasmids. EMBO J 1992 11 3629 3634 1396563 10.1002/j.1460-2075.1992.tb05447.x PMC556822

[B44] Ehrlich SD Berg D, Howe M Illegitimate recombination. Mobile DNA 1989 Washington, DC: American Society for Microbiology 799 832

[B45] Michel B Charlebois RL Illegitimate recombination in bacteria. Organization of the Prokaryotic Genome 1999 Washington DC: ASM Press 129 150

[B46] Della M Palmbos PL Tseng HM Tonkin LM Daley JM Topper LM Pitcher RS Tomkinson AE Wilson TE Doherty AJ Mycobacterial Ku and ligase proteins constitute a two-component NHEJ repair machine. Science 2004 306 683 685 10.1126/science.1099824 15499016

[B47] Weller GR Kysela B Roy R Tonkin LM Scanlan E Della M Devine SK Day JP Wilkinson A d'Adda di Fagagna F Devine KM Bowater RP Jeggo PA Jackson SP Doherty AJ Identification of a DNA nonhomologous end-joining complex in bacteria. Science 2002 297 1686 1689 10.1126/science.1074584 12215643

[B48] Moeller R Stackebrandt E Reitz G Berger T Rettberg P Doherty AJ Horneck G Nicholson WL Role of DNA repair by nonhomologous-end joining in *Bacillus subtilis *spore resistance to extreme dryness, mono- and polychromatic UV, and ionizing radiation. J Bacteriol 2007 189 3306 3311 10.1128/JB.00018-07 17293412 PMC1855867

[B49] Pitcher RS Brissett NC Doherty AJ Nonhomologous end-joining in bacteria: a microbial perspective. Annu Rev Microbiol 2007 61 259 282 10.1146/annurev.micro.61.080706.093354 17506672

[B50] Tsuru T Kawai M Mizutani-Ui Y Uchiyama I Kobayashi I Evolution of paralogous genes: Reconstruction of genome rearrangements through comparison of multiple genomes within *Staphylococcus aureus*. Mol Biol Evol 2006 23 1269 1285 10.1093/molbev/msk013 16601000

[B51] Le Gall T Clermont O Gouriou S Picard B Nassif X Denamur E Tenaillon O Extraintestinal virulence is a coincidental by-product of commensalism in B2 phylogenetic group *Escherichia coli *strains. Mol Biol Evol 2007 24 2373 2384 10.1093/molbev/msm172 17709333

[B52] Lescat M Hoede C Clermont O Garry L Darlu P Tuffery P Denamur E Picard B aes, the gene encoding the esterase B in *Escherichia coli*, is a powerful phylogenetic marker of the species. BMC Microbiol 2009 9 273 10.1186/1471-2180-9-273 20040078 PMC2805673

[B53] Touchon M Hoede C Tenaillon O Barbe V Baeriswyl S Bidet P Bingen E Bonacorsi S Bouchier C Bouvet O Calteau A Chiapello H Clermont O Cruveiller S Danchin A Diard M Dossat C Karoui ME Frapy E Garry L Ghigo JM Gilles AM Johnson J Le Bouguénec C Lescat M Mangenot S Martinez-Jéhanne V Matic I Nassif X Oztas S Organised genome dynamics in the *Escherichia coli *species results in highly diverse adaptive paths. PLoS Genet 2009 5 e1000344 10.1371/journal.pgen.1000344 19165319 PMC2617782

[B54] Deloger M El Karoui M Petit MA A genomic distance based on MUM indicates discontinuity between most bacterial species and genera. J Bacteriol 2009 191 91 99 10.1128/JB.01202-08 18978054 PMC2612450

[B55] MOSAIC http://genome.jouy.inra.fr/mosaic

[B56] Cramton SE Schnell NF Gotz F Bruckner R Identification of a new repetitive element in *Staphylococcus aureus*. Infect Immun 2000 68 2344 2348 10.1128/IAI.68.4.2344-2348.2000 10722640 PMC97424

[B57] Hardy KJ Ussery DW Oppenheim BA Hawkey PM Distribution and characterization of staphylococcal interspersed repeat units (SIRUs) and potential use for strain differentiation. Microbiology 2004 150 4045 4052 10.1099/mic.0.27413-0 15583157

[B58] Perna NT Plunkett G Burland V Mau B Glasner JD Rose DJ Mayhew GF Evans PS Gregor J Kirkpatrick HA Pósfai G Hackett J Klink S Boutin A Shao Y Miller L Grotbeck EJ Davis NW Lim A Dimalanta ET Potamousis KD Apodaca J Anantharaman TS Lin J Yen G Schwartz DC Welch RA Blattner FR Genome sequence of enterohaemorrhagic *Escherichia coli *O157:H7. Nature 2001 409 529 533 10.1038/35054089 11206551

[B59] Volfovsky N Oleksyk TK Cruz KC Truelove AL Stephens RM Smith MW Genome and gene alterations by insertions and deletions in the evolution of human and chimpanzee chromosome 22. BMC Genomics 2009 10 51 10.1186/1471-2164-10-51 19171065 PMC2654908

[B60] Chan SK Hsing M Hormozdiari F Cherkasov A Relationship between insertion/deletion (indel) frequency of proteins and essentiality. BMC Bioinformatics 2007 8 227 10.1186/1471-2105-8-227 17598914 PMC1925122

[B61] Nunes A Borrego MJ Nunes B Florindo C Gomes JP Evolutionary dynamics of ompA, the gene encoding the *Chlamydia trachomatis *key antigen. J Bacteriol 2009 191 7182 7192 10.1128/JB.00895-09 19783629 PMC2786549

[B62] Petersen L Bollback JP Dimmic M Hubisz M Nielsen R Genes under positive selection in *Escherichia coli*. Genome Res 2007 17 1336 1343 10.1101/gr.6254707 17675366 PMC1950902

[B63] Chattopadhyay S Weissman SJ Minin VN Russo TA Dykhuizen DE Sokurenko EV High frequency of hotspot mutations in core genes of *Escherichia coli *due to short-term positive selection. Proc Natl Acad Sci USA 2009 106 12412 12417 10.1073/pnas.0906217106 19617543 PMC2718352

[B64] Shen P Huang HV Homologous recombination in *Escherichia coli *: dependence on substrate length and homology. Genetics 1986 112 441 457 3007275 10.1093/genetics/112.3.441 PMC1202756

[B65] Shimizu H Yamaguchi H Ashizawa Y Kohno Y Asami M Kato J Ikeda H Short-homology-independent illegitimate recombination in *Escherichia coli *: distinct mechanism from short-homology-dependent illegitimate recombination. J Mol Biol 1997 266 297 305 10.1006/jmbi.1996.0794 9047364

[B66] Mau B Glasner JD Darling AE Perna NT Genome-wide detection and analysis of homologous recombination among sequenced strains of *Escherichia coli*. Genome Biol 2006 7 R44 10.1186/gb-2006-7-5-r44 16737554 PMC1779527

[B67] de Vries J Wackernagel W Integration of foreign DNA during natural transformation of *Acinetobacter *sp. by homology-facilitated illegitimate recombination. Proc Natl Acad Sci USA 2002 99 2094 2099 10.1073/pnas.042263399 11854504 PMC122324

[B68] Meier P Wackernagel W Mechanisms of homology-facilitated illegitimate recombination for foreign DNA acquisition in transformable *Pseudomonas stutzeri*. Mol Microbiol 2003 48 1107 1118 10.1046/j.1365-2958.2003.03498.x 12753199

[B69] Prudhomme M Libante V Claverys JP Homologous recombination at the border: insertion-deletions and the trapping of foreign DNA in *Streptococcus pneumoniae*. Proc Natl Acad Sci USA 2002 99 2100 2105 10.1073/pnas.032262999 11854505 PMC122325

[B70] Martinsohn JT Radman M Petit MA The lambda red proteins promote efficient recombination between diverged sequences: implications for bacteriophage genome mosaicism. PLoS Genet 2008 4 e1000065 10.1371/journal.pgen.1000065 18451987 PMC2327257

[B71] Majewski J Cohan FM DNA sequence similarity requirements for interspecific recombination in *Bacillus*. Genetics 1999 153 1525 1533 10581263 10.1093/genetics/153.4.1525 PMC1460850

[B72] Denamur E Lecointre G Darlu P Tenaillon O Acquaviva C Sayada C Sunjevaric I Rothstein R Elion J Taddei F Radman M Matic I Evolutionary implications of the frequent horizontal transfer of mismatch repair genes. Cell 2000 103 711 721 10.1016/S0092-8674(00)00175-6 11114328

[B73] Wang J Gonzalez KD Scaringe WA Tsai K Liu N Gu D Li W Hill KA Sommer SS Evidence for mutation showers. Proc Natl Acad Sci USA 2007 104 8403 8408 10.1073/pnas.0610902104 17485671 PMC1895962

[B74] Drake JW Mutations in clusters and showers. Proc Natl Acad Sci USA 2007 104 8203 8204 10.1073/pnas.0703089104 17495029 PMC1895928

[B75] Yang Y Sterling J Storici F Resnick MA Gordenin DA Hypermutability of damaged single-strand DNA formed at double-strand breaks and uncapped telomeres in yeast *Saccharomyces cerevisiae*. PLoS Genet 2008 4 e1000264 10.1371/journal.pgen.1000264 19023402 PMC2577886

[B76] Kersey P Bower L Morris L Horne A Petryszak R Kanz C Kanapin A Das U Michoud K Phan I Gattiker A Kulikova T Faruque N Duggan K Mclaren P Reimholz B Duret L Penel S Reuter I Apweiler R Integr8 and Genome Reviews: integrated views of complete genomes and proteomes. Nucleic Acids Res 2005 33 D297 302 10.1093/nar/gki039 15608201 PMC539993

[B77] MaGe (Magnifying genomes) Microbial Genome Annotation System https://www.genoscope.cns.fr/agc/mage/wwwpkgdb/MageHome

[B78] Vallenet D Labarre L Rouy Z Barbe V Bocs S Cruveiller S Lajus A Pascal G Scarpelli C Medigue C MaGe: a microbial genome annotation system supported by synteny results. Nucleic Acids Res 2006 34 53 65 10.1093/nar/gkj406 16407324 PMC1326237

[B79] Kurtz S Phillippy A Delcher AL Smoot M Shumway M Antonescu C Salzberg SL Versatile and open software for comparing large genomes. Genome Biol 2004 5 R12 10.1186/gb-2004-5-2-r12 14759262 PMC395750

[B80] *E. coli *O157:H7 Sakai Genome Project http://genome.naist.jp/bacteria/o157/overview.html

[B81] ACLAME: Prophinder http://aclame.ulb.ac.be/Tools/Prophinder/

[B82] CRISPRdb http://crispr.u-psud.fr/crispr/

[B83] Vernikos GS Parkhill J Interpolated variable order motifs for identification of horizontally acquired DNA: revisiting the *Salmonella *pathogenicity islands. Bioinformatics 2006 22 2196 2203 10.1093/bioinformatics/btl369 16837528

[B84] ASAP http://asap.ahabs.wisc.edu/asap/home.php

[B85] BIMEs Table http://www.pasteur.fr/recherche/unites/pmtg/repet/tableauBIMEcoli.html

[B86] The Microorganisms Tandem Repeat Database http://minisatellites.u-psud.fr/GPMS/

[B87] The *Vmatch *large scale sequence analysis software http://www.vmatch.de/

