# Supplementary figures and images for: Small variable segments constitute a major type of diversity of bacterial genomes at the species level
Source: Genome Biol. 2010 Apr 30;11(4):R45. doi: 10.1186/gb-2010-11-4-r45 (PMC2884548; doi:10.1186/gb-2010-11-4-r45)

## *E. coli*, 25 genomes

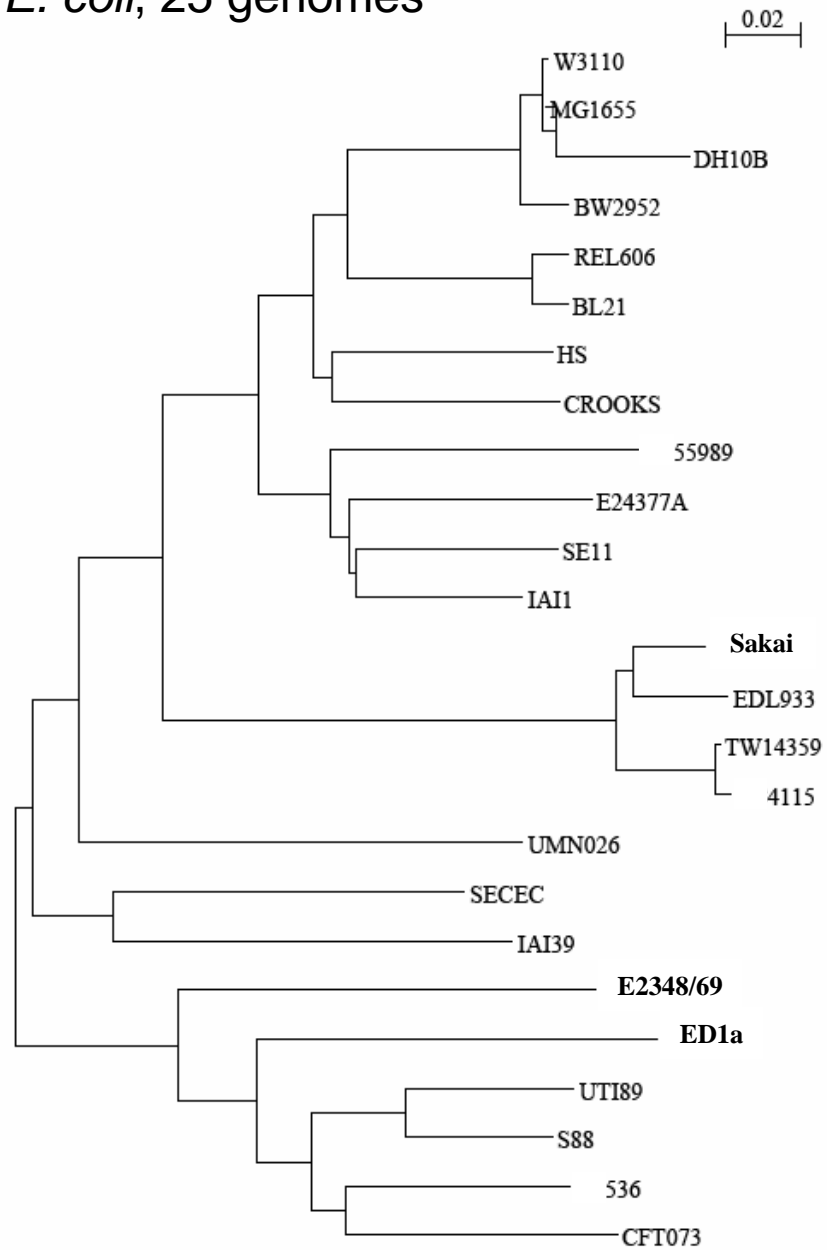

## *S. aureus*, 11 genomes

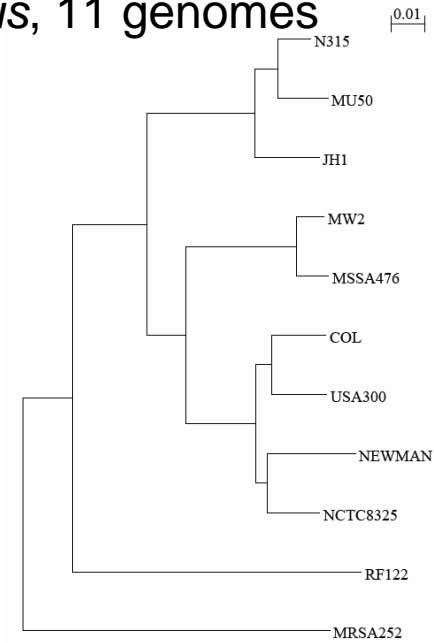

## *S. pyogenes*, 12 genomes

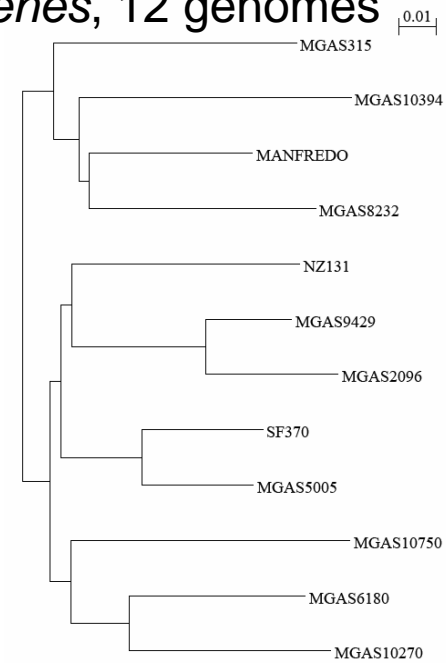

Supplement: Additional file 1 — Neighbor joining trees based on genomic MUMi distances of the strains selected for the maximal genomes alignments. [file gb-2010-11-4-r45-S1.PDF]

## Slide 1
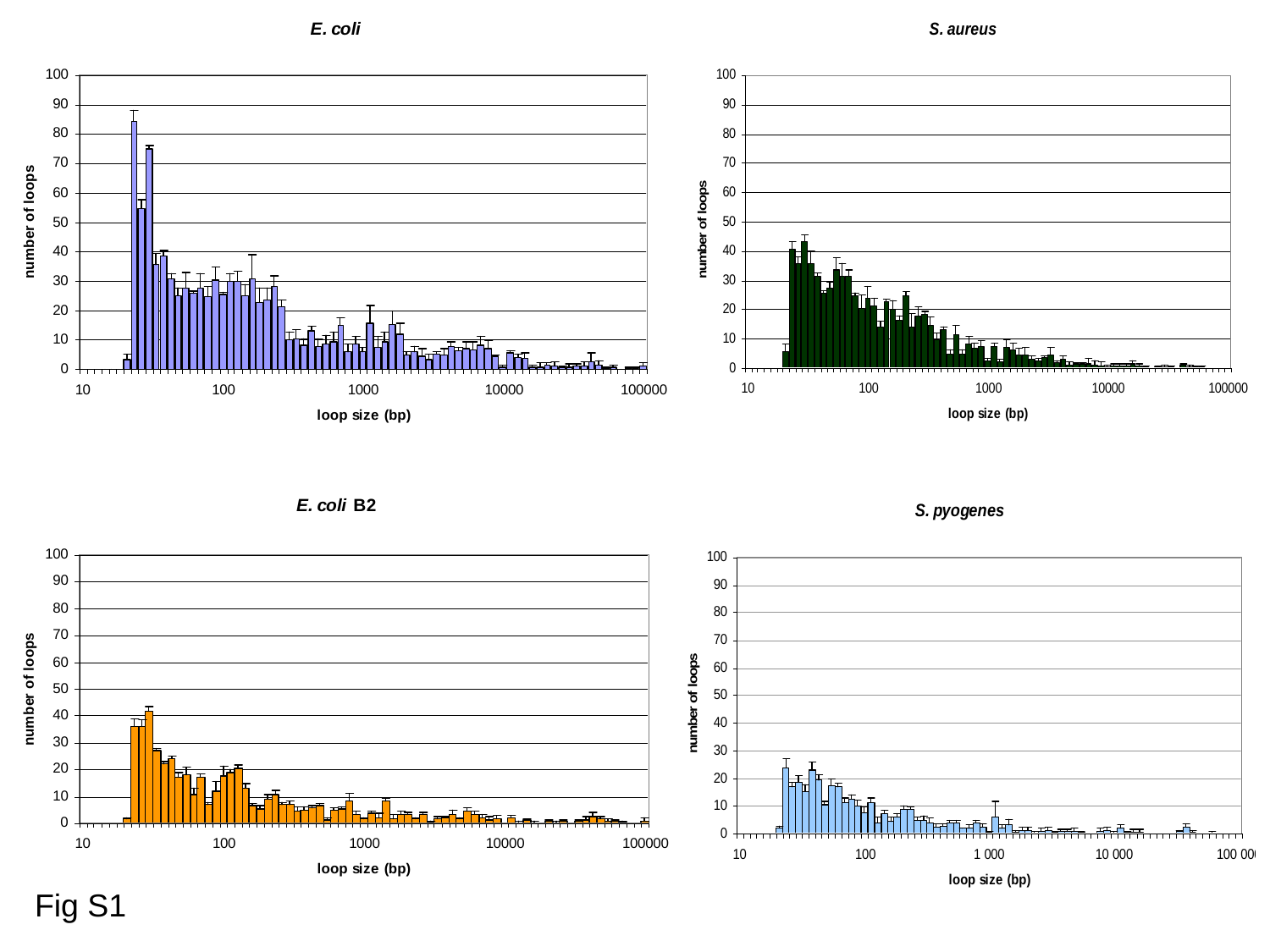

Fig S1

Supplement: Additional file 3 — Distribution of the VS sizes in the five-genome alignments. [file gb-2010-11-4-r45-S3.PPT]
