# Supplementary material for: Small variable segments constitute a major type of diversity of bacterial genomes at the species level
Source: Genome Biol. 2010 Apr 30;11(4):R45. doi: 10.1186/gb-2010-11-4-r45 (PMC2884548; doi:10.1186/gb-2010-11-4-r45)
Supplement: Additional file 2 — Choice of the maximum divergence level for inclusion of ClustalW aligned sequences into the backbone. [file gb-2010-11-4-r45-S2.DOC]

Supplementary Text 1: Choice of the maximum divergence level for inclusion of ClustalW aligned sequences into the backbone.

The last step of most multi-genome aligners (such as MGA) includes a systematic multiple alignment of the remaining gaps (with ClustalW or Muscle), if shorter than the maximal accepted length of these programs (3000 bp and ClustalW are used in the Mosaic database). However, the outputs of this last step are not analysed by MGA (nor MAUVE), and many of the alignments are meaningless, as expected. For inclusion of MGA or MAUVE multiple alignments into the Mosaic database, a post-treatment step on the raw ClustalW results is therefore requested, to define, among the outputs, those in which the alignment reflects common ancestry, from those where different pieces of DNA are forced into an alignment. Each alignment is scanned and two parameters are taken into consideration to decide inclusion into the backbone: the number of consecutive gaps (≤20, see Methods), and the pairwise percentage identity of all aligned pairs included into the alignment.

To determine experimentally which threshold **T** to use for the pairwise percentage identity, a reference *E. coli* MGA alignment of strains MG1655 and Sakaï was post-treated using values between 70% and 95% for **T**, and the effect of this parameter on the average pairwise identity of the backbone, *π *_back_, was measured. A plot of *π *_back_ as a function of **T** is shown Fig.1. A bi-phasic curve is obtained, with nearly constant *π*_back_ values around 98.3% in the 70-76% threshold range, and increasing values of *π *_back_ up to 98.8% when **T** is above 76%.

Figure 1: Effect of the parameter T, *ie* the minimal pairwise identity in the ClustalW alignment to decide inclusion into the backbone, on the global percentage of identity of the backbone in the MG1655-Sakaï whole genome alignment with MGA.

The point of inflection of this curve, at 76%, was chosen for **T**, for the following reason: The *π* _back_ value can be estimated independently by ANI, the average nucleotide identity (Goris, Konstantidinis, Klappenbach, Coenye, Vandamme, Tiedje, 2007, *Int. J. Sys. Evol. Microbiol.* 57, 81-91). The ANI value of the MG1655-Sakaï pair of genomes is 97.9% (calculated as in Goris et al, the average of the MG-Sakaï value and the Sakaï-MG value is given). It appears therefore that the lower *π *_back_ value, found with the 70-76% range is closer to the ANI estimate. Because 76% identity is already low as compared to the overall global percentage of identity of the backbone, we decided to use 76%, the upper limit of the 70-76% range, as the **T** value.
