# Supplementary material for: Small variable segments constitute a major type of diversity of bacterial genomes at the species level
Source: Genome Biol. 2010 Apr 30;11(4):R45. doi: 10.1186/gb-2010-11-4-r45 (PMC2884548; doi:10.1186/gb-2010-11-4-r45)
Supplement: Additional file 14 — Loci occupancy in the five-genome alignments. [file gb-2010-11-4-r45-S14.DOC]

**Table S11** Occupancy of the microdiversity loci in the four alignments

|  | *E. coli* | *E. coli* B2 | *S. aureus* | *S. pyogenes* |
| --- | --- | --- | --- | --- |
| Total number of Loci | 640 | 370 | 556 | 250 |
| % of loci with occupancy 1 | 7% | 9% | 9% | 5% |
| % of loci with occupancy 2 | 3% | 5% | 3% | 3% |
| % of loci with occupancy 3 | 2% | 3% | 4% | 4% |
| % of loci with occupancy 4 | 8% | 8% | 6% | 8% |
| % of loci with occupancy 5 | 79% | 75% | 78% | 80% |
